# Potential Application of Some Lamiaceae Species in the Management of Diabetes

**DOI:** 10.3390/plants10020279

**Published:** 2021-02-01

**Authors:** Ninon G.E.R. Etsassala, Ahmed A. Hussein, Felix Nchu

**Affiliations:** 1 Department of Horticultural Sciences, Faculty of Applied Sciences, Cape Peninsula University of Technology, Symphony Road, Bellville 7535, South Africa; 3415216@myuwc.ac.za; 2 Chemistry Department, Cape Peninsula University of Technology, Bellville Campus, Symphony Road, Bellville 7535, South Africa; mohammedam@cput.ac.za

**Keywords:** Lamiaceae species, diabetes mellitus, oxidative stress, secondary metabolites

## Abstract

Diabetes is one of the most dangerous metabolic disorders, with high rates of mortality worldwide. Since ancient times, medicinal plants have been used in traditional medicine to treat many diseases, including diabetes and its related complications. Plants are widely accepted, affordable, and perceived to have minimal adverse side effects. The Lamiaceae family is a potential source of therapeutic agents for the management of metabolic disorders, including diabetes. Hence, this review paper summarizes the antidiabetic use of Lamiaceae species in folk medicine globally. Furthermore, we present the antidiabetic activities and phytochemical constituents of twenty-three (23) Lamiaceae species and the antidiabetic activity of some notable chemical constituents isolated from some of these Lamiaceae species.

## 1. Introduction

Diabetes mellitus (DM) is one of the most dangerous metabolic disorders, causing high mortality rates worldwide [1]. It is well known that insulin plays a crucial role in glucose homeostasis, as it activates the transport of blood glucose inside the skeletal muscle [2]. However, insulin resistance in target tissues and the shortage of insulin production from pancreatic β-cells are the principal attributes of type 2 diabetes. Additionally, type 2 DM characterization also encompasses a decrease in peripheral glucose uptake within the muscle, adipose, or liver cells and an increase in endogenous glucose secretion, causing increased blood glucose concentration [3,4,5]. Therefore, agents with the ability to activate glucose uptake in these tissues can ameliorate insulin resistance and treat diabetes [6]. Many synthetic antidiabetic drugs such as acarbose, sulfonylurea, miglitol, metformin, and thiazolidinedione are already present in the market. However, their effectiveness is restricted because of their high cost and adverse side effects [7,8], which incentivize the development of powerful natural antidiabetic products/drugs with minimal adverse side effects.

Lamiaceae, commonly known as the mint family, is a cosmopolitan flowering plant family with approximately 7136 species assigned to 236 genera. Most of the species are herbaceous or shrubby, and trees are scarce. The most well-known genus is *Salvia* (900), followed by *Scutellaria* (360), *Stachys* (300), *Plectranthus* (300), *Hyptis* (280), *Teucrium* (250), *Vitex* (250), *Thymus* (220), *Nepeta* (200), and *Clerodendrum* (150) [9].

The family possesses significant economic value, as it contains some horticultural species and culinary herbs, such as rosemary, salvia, ocimum, and leonotis. They are well reputed because of the high level of aromatic compounds within their leaves, flowers, and essential oils. These compounds are pharmacologically active terpenoids that play vital roles in developing new bioactive products within the cosmeceuticals, nutraceuticals, and pharmaceutical industries [10]. People use many species in this family for culinary, fragrance, flavour, and aromatherapy purposes.

Additionally, Lamiaceae species have antispasmodic, antiseptic, antimicrobial, calmative, and antidiabetic activities [11]. Lamiaceae species have been used as antidiabetic agents since time immemorial for their curative abilities within the folk medicine of divergent communities, regions, and tribes in the African sub-region and worldwide [12]. This study reviews the traditional use of the Lamiaceae species to treat diabetes globally and reports on the isolated antidiabetic compounds and their pharmacological actions from Lamiaceae species.

## 2. Method

This review summarized previous research works (1986–2020) conducted on plants of the Lamiaceae species that have been traditionally used to treat diabetes worldwide and the antidiabetic bioactivities of their extracts and pure compounds. The keywords “Lamiaceae species” and “treatment of diabetes” as well as scientific databases such as SciFinder, Science Direct, and Google Scholar were used to source primary and secondary data for this review. Additionally, we used a database of plants (http://www.theplantlist.org/) to check the scientific names of plants and their authority. Furthermore, Quantum GIS was used to produce a map of the traditional use of the Lamiaceae species to manage diabetes in different countries and regions of the world.

## 3. Lamiaceae Species Used for Diabetes

### 3.1. Ajuga iva (L.) Schreb

#### 3.1.1. Traditional Uses

*Ajuga iva* is widely distributed in the South European and North African countries [13].

In Morocco and Algeria, a decoction of *A. iva* is utilized in traditional medicine to treat different kinds of diseases, including diabetes. Additionally, many Moroccans use its decoction to alleviate diabetes [13,14,15].

#### 3.1.2. Phytochemical Constituents

The phytochemical investigation of the extracts derived from different parts of *A. iva* showed the presence of diverse classes of bioactive secondary metabolites such as flavonoids, steroids, terpenoids, and fatty acids [16]. Five terpenoids such as ivain I-V were isolated from the diethyl ether extract of the whole plant of *A. iva*, in addition to 6-desoxyharpagide, 3-8-*O*-acetylharpagide, 8-*O*-acetylharpagide, and harpagide. Flavonoids, such as apigenin hexoside-pentoside, apigenin dihexoside, apigenin 7-*O*-neohesperidoside, naringenin-hexoside, apigenin 7-*O*-glucoside, and naringenin-7-*O*-α-L-rhamnopyranosyl, were reported from *A. iva* [13,17]. Other compounds, including 5-caffeoylquinic acid, apigenin 6,8-di-*C*-glucoside, 3,5-dicaffeoylquinic acid, and naringin were predominantly found in the infusion of *A. iva* [18].

#### 3.1.3. Antidiabetic Activity

In vitro and in vivo biological investigations revealed that the methanolic extract of *A. iva* has antidiabetic activity [16,19]. *A. iva* possesses hypoglycaemic and hypolipidemic activities [19]. The bio-evaluation of the alpha-amylase and alpha-glucosidase inhibitory activities of the aqueous and methanolic extracts of the aerial parts of *A. iva* showed a good inhibition of alpha-amylase, with IC_50_ values of 0.210 ± 0.003 and 0.180 ± 0.005 μg/mL, as well as of alpha-glucosidase, with IC_50_ values of 0.172 ± 0.012 and 0.130 ± 0.008 μg/mL, respectively [17].

The whole plant of *A. iva* has been reported to increase the hepatic glycogen concentration and prevent diabetic complications in the kidneys, pancreas, and liver. Additionally, the extract of *A. iva* showed a preventive effect against the deleterious effects of diabetes on oxidative stress [18]. The administration of the extract of *A. iva* significantly reduced the plasma glucose concentration and consequently resulted in the rapid normalization of glucose levels in diabetic animals [18]. The aqueous extract of *A. iva* significantly decreased the plasma glucose level in STZ-diabetic rats, with no effect on insulin production. Additionally, *A. iva* upgraded the glycaemic value (41%) in hyperglycaemic rats and lessened the glycosylated haemoglobin (HbA1c) [19]. The lyophilized aqueous extract of *A. iva* (whole plant) displayed significant hypoglycaemic activity and was relatively non-toxic to normal (normoglycemic) and streptozotocin (STZ)-diabetic rats [19]. An aqueous extract of the whole plant of *A. iva* showed hypolipidemic and hypoglycaemic effects in both normoglycemic and diabetic rats [19]. Additionally, the aqueous extract of *A. iva* is a rich source of phytoecdysteroids, which are potential therapeutic candidates for alloxan-induced diabetic male albino rats [20].

*A. iva* aqueous extract demonstrated significant hypolipidemic activity after a single dose and repeated treatments on STZ-diabetic rats [15].

### 3.2. Ballota nigra L.

#### 3.2.1. Traditional Uses

*Ballota nigra* is native to the Mediterranean region and predominantly found in Europe and Asia [21].

People in many parts of Turkey use the aerial parts of *B. nigra* traditionally to treat haemorrhoids, wounds, ulcers, animal bites, sores, flu, colds, and flatulence, and as an antiseptic for inflamed skin, wounds, burns, and diabetes mellitus [21,22].

#### 3.2.2. Phytochemical Constituents

Numerous compounds such as 7α-acetoxymarrubiin, ballonigrin, ballotenol, ballotinone, dehydrohispanolone (hispanone), ballonigrin, ballotenol, ballotinone, marrubiin, preleosibirin, hydroxyballonigrolide, siderol, dehydrohispanolone, apigenin-7-glucoside, luteolin-7-glucosyl-lactate, luteolin-7-lactate, ladanein, vicenin-2, tangeretin, caffeic, caffeoylmalic, and chlorogenic acids were isolated from *B. nigra* [21].

#### 3.2.3. Antidiabetic Activity

A 70% ethanol extract of *Ballota nigra* has been reported to possess hypoglycaemic, insulin-releasing, and cholesterol-lowering effects in rats [22].

### 3.3. Becium grandiflorum (Lam.) Pic. Serm.

#### 3.3.1. Traditional Uses

*Becium grandiflorum* is endemic to Ethiopia and Eretria. It occurs in Kenya and Tanzania [23].

The fresh leaves of *B. grandiflorum* are traditionally used to treat many ailments such as malaria, bacterial infections, diabetes mellitus, wound healing, influenza, respiratory depression, and inflammatory disorders [24].

#### 3.3.2. Phytochemical Constituents

The predominant vacuolar flavonoid of *B. grandiflorum* is the 8-*O*-glucoside of isothymusin, while the significant external flavonoids are isothymusin and cirsimaritin [23].

#### 3.3.3. Antidiabetic Activity

The hydroalcoholic extract of *B. grandiflorum* has been reported to exhibit significant antihyperglycemic activity (*p* 0.05) in STZ-induced diabetic mice. It also showed a considerable amelioration in oral glucose tolerance and body weight, which justified this species’ potential usage in managing diabetes mellitus complications in Ethiopian folk medicine [25].

### 3.4. Calamintha officinalis Moench

#### 3.4.1. Traditional Uses

*Calamintha officinalis* is native to the northern part of Iran [26]. It is widely distributed in Southern and Central Europe, Western Asia, and North Africa [27].

Different parts of *C. officinalis* (stem, leaves, and seeds) are used to treat different diseases, including lowering the blood glucose level in diabetic patients [28,29].

#### 3.4.2. Phytochemical Constituents

The phytochemical studies of the aerial part of *C. officinalis* revealed the presence of polyphenolic compounds, such as chlorogenic, caffeic, hydroxycinnamic, and rosmarinic acids [30].

#### 3.4.3. Antidiabetic Activity

The bio-evaluation of the aqueous extract of *C. officinalis* showed significant hypoglycaemic activity in normal and streptozotocin-induced diabetic rats without modifying the concentrations of basal plasma insulin [31]. Additionally, the aqueous extract of *C. officinalis* demonstrated remarkable hypoglycaemic activity in normal and STZ diabetic rats without influencing the basal plasma insulin concentrations [30]. The antidiabetic and antioxidant activities of the crude extract and its isolates (rosmarinic and caffeic acids) from the aerial parts of *C. officinalis* revealed that both rosmarinic and caffeic acids are prominent natural agents for controlling diabetes [30].

### 3.5. Coleus forskohlii (Willd.) Briq

#### 3.5.1. Traditional Uses

*Coleus forskohlii* is native to India. It is widely distributed in Nepal, Thailand, and Sri Lanka [32].

Across West Africa and India, people use Coleus traditionally for the treatment of diabetes [33]. Historically, communities use *Coleus* to treat hypertension, eczema, colic, congestive, heart failure, painful urination, respiratory problems, sleeplessness, and convulsions [34,35,36].

#### 3.5.2. Phytochemical Constituents

Forskolin was the first labdane diterpene isolated from the root of *Coleus* in 1974 [32]. Additionally, forskolin derivatives such as forskolin E, F, G, and H were isolated from the same source [37]. The phytochemistry of *Coleus* is mainly composed of diterpenes [35]. Approximately 20 constituents have been found in different parts of the plant, whereas forskolin and coleonols are the most predominant phytochemicals of the root [34]. Other minor diterpenes such as 9-deoxyforskolin, deactylforskolin, 9-dideoxy-7-deacetylforskolin, and 1,9-dideoxy-7-deacetylforskolin have been isolated from the root extract. Additionally, 1,6-diacetoxy-9-deoxyforskolin, forskolin I, forskolin J, and forskolin L were isolated from Chinese *Coleus* [37]. Two more diterpenoids, such as 6-acetyl-1,9-dideoxy forskolin and 6-acetyl-1-deoxyforskolin, were also reported. Another three new minor labdane diterpene glycosides, forskoditerpenoside C-E, and a novel labdane diterpene forskoditerpene A were isolated from the ethanolic extract of the whole plant [37]. Coleonol E and F were reported from Indian *Coleus*. Coleol and coleosol were isolated from the roots. Coleon O and S and plectrin were reported from the leaves of Kenyan *Coleus* [35]. The presence of 3-hydroxyisoforskolin and 3-hydroxy forskolin was also mentioned in the same source [37].

#### 3.5.3. Antidiabetic Activity

The leaves of *Coleus* have been reported to have a wide range of pharmaceutical applications, including in diabetes and weight loss [34]. The extract of *Coleus* has been reported to attenuate/reduce the hypoglycaemic action of tolbutamide via a hepatic CYP2C-mediated mechanism [38]. Forskolin, the main predominant constituent of *C*. *forskohlii,* has been reported to stimulate glucose-induced insulin secretion in the in vitro model [37,39].

### 3.6. Hyptis suaveolens (L.) Poit

#### 3.6.1. Traditional Uses

*Hyptis suaveolens* is native to tropical America. It is widely distributed in the Northern Territory and Queensland of Australia, China, Indonesia, French Polynesia, the Federated States of Micronesia, the Niue Islands, Guam, the Hawaiian Islands of the USA, and West and Central Africa [40].

*H. suaveolens* is traditionally used to treat diabetes mellitus, eczema, fever, cancer, and headache [41,42].

#### 3.6.2. Phytochemical Constituents

Diterpenes (suaveolic acid, suaveolol, and methyl suaveolate), phenolic acids (rosmarinic acid and methyl rosmarinate), and triterpenes (oleanolic and ursolic acids) were isolated from *H. suaveolens* [40].

#### 3.6.3. Antidiabetic Activity

The 50% aqueous ethanolic extract of *H. suaveolens* has been reported to possess significant antihyperglycemic activity in streptozotocin-induced diabetic rats and decrease the cholesterol and triglyceride levels in a significant manner [43]. The aerial part of *H. suaveolens* has been reported to possess antidiabetic and antioxidant properties [41].

### 3.7. Lavandula angustifolia Mill

#### 3.7.1. Traditional Uses

*Lavandula angustifolia* is native to the northern region of Jordan and the Mediterranean region (France, Spain, and Italy) [44].

*L. angustifolia* has been used in Jordanian folk medicine since ancient times in the management of diabetes [45,46,47].

#### 3.7.2. Phytochemical Constituents

*L. angustifolia* is a rich source of phenolic derivatives, especially rosmarinic and gallic acids [48]. Other phenolic compounds, such as lavandufurandiol, lavandunat, lavandupyrones A and B, lavandufluoren, lavandudiphenyls A and B, ethyl 3-phenylpropionate, 4-(1-hydroxy-1-methylethyl) benzoic acid, methyl 3-(3,4-dihydroxyphenyl)propanoate, and isosalvianolic acid, were isolated from the ethyl acetate extract of *L. angustifolia* [49].

#### 3.7.3. Antidiabetic Activity

A bio-evaluation of the methanolic extract of *L. angustifolia* regarding the management of diabetic dyslipidaemia demonstrated that *L. angustifolia* can inhibit HSL and PL activities in a dose-dependent manner, with IC_50_ values of 175.5 and 56.5 µg/mL, respectively. The inhibitory activity demonstrated by *L. angustifolia* could be attributed to the presence of rosmarinic acid with IC_50_ values of 125.2 and 51.5 µg/mL for PL and HSL, respectively, and gallic acid with IC_50_ values of 10.1 and 14.5 µg/mL for PL and HSL, respectively, which are the major compounds of *L. angustifolia* [45].

### 3.8. Lavandula dentata L.

#### 3.8.1. Traditional Uses

*Lavandula dentata* occurs in the Mediterranean and Saharan regions [50].

*L. dentata* is traditionally used in various parts of the world to treat gastrointestinal, diabetes mellitus, nervous, and rheumatic ailments [51].

#### 3.8.2. Phytochemical Constituents

Phytochemical studies showed that triterpenoids (ursolic acid), flavonoids (luteolin), and coumarins (umbelliferone) were the main phytochemical constituents of the aerial parts of *L. dentata* [52]. Additionally, three classes of secondary metabolites, such as phenolic compounds, terpenes, and alkaloids, are predominantly found in *L. dentata* [53].

#### 3.8.3. Antidiabetic Activity

*L. dentata* has been reported to exhibit hypolipidemic, antioxidant, and hypoglycaemic activities. It has also been reported to reduce blood sugar levels (*p* 0.05) [54].

### 3.9. Lavandula multifida L.

#### 3.9.1. Traditional Uses

*Lavandula multifida* is native to south-western Europe, the Mediterranean, and North Africa (from Morocco to Egypt) [55].

*L. multifida* is traditionally used to treat headaches, depression, migraine, stress, and diabetes [56,57].

#### 3.9.2. Phytochemical Constituents

Phytochemical studies of the leaves of *L. multifida* led to the isolation of luteolin-7-*O*-glycoside, isoscutellarin-8-*O*-glycosides, and hypolaetin-8-*O*-glycosides [56]. Other phytochemical studies revealed the presence of 2*α*,3*β*-dihydroxy-olean-12-en-28-oic acid (maslinic acid), pimarane diterpenes (15*S*,16-dihydroxy-7-oxopimar-8(9)-ene and 15,16,17-trihydroxy-7-oxopimar-8(9)-ene), pimarane, *iso*-pimarane diterpenes (15*S*,16-dihydroxyisopimar-8(9)-ene (glutinosin), 15,16-dihydroxy-7,11-dioxopimar-8(9)-ene, and 15,16,17-trihydroxypimar-8(9)-ene), and carvacrol [56]. Furthermore, numerous compounds such as glutinosin, 15,16,17-trihydroxy-7-oxopimar-8(9)-ene, 15,16-dihydroxy-7,11-dioxopimar-8(9)-ene, and 15,16,17-trihydroxypimar-8(9)-ene were also isolated [55].

#### 3.9.3. Antidiabetic Activity

*L. multifida* has been reported to possess antioxidant and antihypolipidemic activities [58]. Additionally, it has been also reported for its potent hypoglycaemic activity [55].

### 3.10. Lavandula stoechas L.

#### 3.10.1. Traditional Uses

*Lavandula stoechas* is widely distributed in Morocco, Tunisia, Algeria, Spain, France, Greece, Italy, Turkey, Iran, and Saudi Arabia and around the Mediterranean basin [59].

*L. stoechas* is used in traditional Tunisian medicine to treat depression, headaches, and diabetes [60].

#### 3.10.2. Phytochemical Constituents

The main phytochemical constituents of the leaves of *L. stoechas* are flavonoids, flavone glycosides, and flavone 7-omonoglycosides. Numerous compounds have occurred in the aerial parts of *L. stoechas* extracts. These include ursolic, vergatic, and oleanolic acids; α-amyrin, α-amyrin acetate, β-sitosterol, lupeol, erythrodiol, vitexin, acacetin; two longipinane derivatives (longipin-2-ene-7β,9α-diol-1-one and longipin-2-ene-7β,9α-diol-1-one-9-monoacetate); protocatechuic, chlorogenic, caffeic, rosmarinic, and ferulic acids; and pinobanksin, quercetin, pinocembrin, and luteolin [59]. The presence of polyphenols, flavonoids, tannins, saponins, sterols, triterpenes, and cardiac glycosides was found in the ethyl acetate extract of *L. stoechas* [61].

#### 3.10.3. Antidiabetic Activity

*L. stoechas* has been reported to reduce blood sugar levels [60,62]. The aerial parts of *L. stoechas* effectively protect against increases in the blood glucose level, and a decrease in the antioxidant activities was observed [60].

### 3.11. Leonotis leonurus (L.) R.Br

#### 3.11.1. Traditional Uses

*Leonotis leonurus* is native to Southern Africa. It is widely distributed in the Eastern and Western Cape, Kwazulu-Natal, and Mpumalanga provinces [63].

*L. leonurus* (leaves and stems) is traditionally used in South Africa to treat diabetes, coughs, colds, influenza, chest infections, hypertension, eczema, epilepsy, menstruation delayed, intestinal worms, and constipation [63,64]. The tea prepared from the whole plant is utilized for piles, arthritis, bladder and kidney diseases, cancer, obesity, and rheumatism [65].

#### 3.11.2. Phytochemical Constituents

The phytochemical investigation of *L. leonurus* showed the presence of sterols, flavonoids, diterpenes, triterpenoid, tannins, carbohydrates, quinines, and alkaloids. Flavonoids such as apigenin 8-*C*-glucoside, apigenin 6-*C*-arabinoside-8-*C*-glucoside, apigenin 7-*O*-glucoside, luteolin, luteolin 7-*O*-glucoside-3-methyl ether, luteolin 7-*O*-glucoside, apigenin 7-*O*-(6-*O*-*p*-coumaroyl)-glucoside luteolin 3-methyl ether, 6-methoxyluteolin-4-methyl ether, and apigenin were isolated [63]. Diterpenoids such as marrubin and leoleorin K, L, M, and N have been reported from the leaves [65].

#### 3.11.3. Antidiabetic Activity

*L. leonurus* has been reported to lower the blood glucose level in streptozotocin-induced diabetic rats. Additionally, *L. leonurus*’ aqueous extract has antihyperglycaemic and antilipidemic activities. Its aqueous leaf extract induced a significant (*p* 0.05–0.001) hypoglycaemic effect in rats, which was ascribed to different diterpenoids, polyphenolics, flavonoids, and other phytochemical constituents of the plant extract [66].

### 3.12. Leonotis nepetifolia (L.) R.Br

#### 3.12.1. Traditional Uses

*Leonotis nepetifolia* is native to tropical and subtropical Africa and has been naturalized worldwide [67].

*L. nepetifolia* is traditionally used to treat kidney diseases, rheumatism, dysmenorrhea, bronchial asthma, diarrhoea, fever, influenza, colds, and coughs as well as adult-onset type-2 diabetes mellitus [68]. In India, the plant is used for skin problems, malaria, and rheumatism. The plant is also used to treat asthma and epilepsy in South Africa [69].

#### 3.12.2. Phytochemical Constituents

The phytochemical investigation of the whole plant of *L. nepetifolia* led to the isolation of 8β,17:9,13-diepoxylabdane-16,15:19,6β-dilactone, 4,6,7-trimethoxy-5-methylchromen-2-one, nepetaefolinol, and leonotinin. The leaves contain labdane diterpenes such as nepetaefolin as well as methoxynepetaefolin, *bis*-spirolabdane, leonepetaefolins A–E, 15-*epi*-leonepetaefolins A–E, and flavonoids (apigenin and cirsiliol) [70,71].

#### 3.12.3. Antidiabetic Activity

The bio-evaluation of the ethanolic extract of the whole plant of *L. nepetifolia* exhibited a potent antidiabetic activity in diabetic rats [72].

### 3.13. Marrubium vulgare L.

#### 3.13.1. Traditional Uses

*Marrubium vulgare* is native to the Mediterranean Sea region and widely distributed in many temperate regions of North Africa, Asia, and Europe [73].

*M. vulgare* is used in Morocco, Mexico, and Algeria in the treatment of diabetes mellitus [74,75].

#### 3.13.2. Phytochemical Constituents

*Marrubium vulgare* is a rich source of polysaccharides, amino acids, tannins, flavonoids, phenols, terpenes, alkaloids, and steroids [76]. Numerous phytochemicals such as flavonoids (luteolin, ladanein, apigenin, quercetin, chrysoeriol, isoquercitrin, and vitexin), diterpenes (marrubiin and related compounds), phenylpropanoid esters (acteoside (or verbascoside), forsythoside B, ballotetroside, arenarioside, marruboside, alyssonoide, and marruboside), tannins (catechin, epicatechin, proanthocyanidins, and condensed tannins), and sterols have been isolated from *M. vulgare* [77]. Several flavonoid glycosides and labdane diterpenoids have been isolated from the same source. The phytochemical investigation of *M. vulgare* produced a labdane diterpenoid, a glycosidic peregrinol, and a flavonoid derivative, in addition to apigenin-4’-*O*-(6’’-*O*-*p*-coumaroyl)-β-D-glucopyranoside, polyodonine, and 4’,5,7- trihydroxyflavone [78,79].

#### 3.13.3. Antidiabetic Activity

Scientific studies on *M. vulgare* have demonstrated through in vivo research the hypoglycaemic effect of *M. vulgare*, which supports its traditional use in controlling diabetes mellitus [80]. *M. vulgare* has been reported to possess hypoglycaemic and antioxidant activities. The 80% ethanolic extract of *M. vulgare* showed a moderate alpha-glucosidase inhibitory activity, with an IC_50_ value of 12.66 µg/mL [78,81]. The methanolic extract exhibited a considerable decrease in blood glucose and a significant increase in plasma insulin and tissue glycogen contents [82]. The administration of an infusion from the aerial parts of *M. vulgare* significantly decreased the blood glucose level in a dose-dependent manner in alloxan-induced diabetic rats [83]. The ethanolic extract from the root considerably suppressed the increase in the plasma glucose level in healthy rats [83]. Moreover, *M. vulgare* shows an antidiabetic effect by suppressing the carbohydrate absorption from the intestine and thereby reducing the postprandial increase in the blood glucose level [74]. The oral administration of the aqueous extract induced significant antidiabetic and antihyperlipidemic dose-dependent effects in treated animals [75]. *M. vulgare* significantly lessen the blood glucose level, pancreatic levels of interferon-gamma and nitric oxide, total cholesterol, low-density lipoprotein (LDL), and very LDL cholesterol and triglycerides compared with diabetic mice [79]. The methanolic extract was found to have PPARγ agonist activity in a luciferase reporter assay. PPARγ adjusts the glucose and lipid metabolism and its synthetic agonists such as pioglitazone ameliorate insulin resistance, thus it is clinically employed for diabetes therapy [84].

### 3.14. Ocimum gratissimum L.

#### 3.14.1. Traditional Uses

*Ocimum gratissimum* is native to Asia and South Africa [85].

*O. gratissimum* is widely used in Africa and Asia for treating diabetic symptoms. It is traditionally utilized in Western Africa and Nigeria as a febrifuge, antimalarial, anticonvulsant, antimicrobial, and antioxidant agent and for the treatment of high fever, epilepsy, diarrhoea, mental illness, and diabetes mellitus [86]. The plant is also used in Africa to treat bacterial and fungal infections, fever, colds, stomach upset, haemorrhoids, catarrh, and diabetes [87,88,89,90].

#### 3.14.2. Phytochemical Constituents

Numerous classes of compounds such as flavonoids, polyphenols, quinones, coumarins, and catechins were found in the aqueous extract of *O. gratissimum* [87,91]. Oleanolic acid, cirsimaritin, xanthomicrol, kaempferol 3-rutinoside, rutin, vicenin-2, luteolin 5-*O* and 7-*O*-glucosides, isothymusin, quercetin 3-*O*-glucoside, vitexin, apigenin 7-*O*-glucoside, luteolin, isovitexin, caffeic acid, rosmarinic, chlorogenic, chicoric acids, and nevadensin have been reported [92].

#### 3.14.3. Antidiabetic Activity

The methanolic and aqueous extracts of the leaves showed hypoglycaemic activity. Additionally, the aqueous extract at the dose of 500 mg/kg significantly decreased the blood glucose level (*p* 0.05) of diabetic rats by 81.3% after 24 h of extract administration [93]. The leaf extract was reported to have antidiabetic activity in streptozocin-induced diabetic rats [88]. *O. gratissimum* decreased the baseline blood glucose levels in normal and alloxan-induced rats [94]. The leaf extract showed a potential plasma glucose lowering effect [95].

The aqueous extract showed anti-hyperglycaemic and antioxidant potentials. The hypoglycaemic effect of the methanolic extracts showed a decrease in the blood glucose level of 69% and 56% for alloxan-induced diabetic and normal rats, respectively [96].

### 3.15. Ocimum sanctum L.

#### 3.15.1. Traditional Uses

*Ocimum sanctum* is native to India and is widely distributed in Australia, Malaysia, West Africa, and Arab countries [97].

*O. sanctum* is used worldwide to reduce the risk factors associated with several disorders, including hypoglycaemia [98].

#### 3.15.2. Phytochemical Constituents

Rosmarinic acid, apigenin, propanoic acid, isothymusin, cirsimaritin, orientin, vicenin, and isothymonin have been found from the leaves [99].

#### 3.15.3. Antidiabetic Activity

The aqueous suspension considerably decreases the blood glucose level (*P* 0.0001) and oxidative stress with a significant increase in glycogen and protein in diabetic rats [96,98]. A 70% ethanol extract of the leaves of *O. sanctum* has been reported to significantly decrease the blood glucose level in both normal and streptozotocin-induced diabetic rats [96]. In vivo studies of the ethanolic extract have also shown a decrease in the blood glucose level and an increase in the plasma insulin activity in type 2 diabetes mellitus. Another study showed a significant decrease in diabetic symptoms (polyphagia, polydipsia, and tiredness) in type 2 diabetic patients who consumed the leaf powders of *O. sanctum* [96]. Additionally, the ethanol extract activates insulin production from the perfused pancreas, isolated islets, and clonal pancreatic cells [100]. The leaf extracts of *O. sanctum* have been shown to have anti-hyperglycaemic effects by increasing the insulin secretion from isolated islets, the perfused pancreas, and clonal pancreatic β–cells [96,101].

### 3.16. Ocimum basilicum L.

#### 3.16.1. Traditional Uses

*Ocimum basilicum* is native to Africa and Asia [96,102].

Basil improves digestion and is also suitable for curing epistaxis when mixed with camphor. The infusion of *O. basilicum* is useful in cephalagia, fever, coughs, gouty joints, otitis, snake bites, stomach problems, and gout and is given internally to treat cystitis, nephritis, and internal piles. The infusion of basil seed is used to treat gonorrhoea, chronic dysentery, diarrhoea, and diabetes mellitus [103,104].

#### 3.16.2. Phytochemical Constituents

The phytochemical analysis of the aqueous extract of *O. basilicum* showed the presence of tannins, saponins, and cardiac glycosides [103]. Phenolic acids, caffeic acid derivatives, and flavonol-glycosides are the main constituents found in *O. basilicum* [103].

#### 3.16.3. Antidiabetic Activity

The aqueous extract significantly lowered both plasma triglycerides (TG) and cholesterol in acute hyperlipidaemia induced by Triton WR-1339 in rats [105]. The aqueous extract of the whole plant exhibited a hypoglycaemic effect in normal and streptozotocin diabetic rats [106]. Furthermore, the methanol-dichloromethane extract of the leaves has anti-hyperglycaemic effects [96]. The extracts have been reported to possess different pharmacological effects, including blood glucose-lowering and hepatoprotective properties [107]. The extract of the aerial parts possessed antidiabetic effects, which might be mediated by limiting glucose absorption through the inhibition of carbohydrate metabolizing enzymes and the enhancement of hepatic glucose mobilization [107].

The extract demonstrated significant dose-dependent inhibition against rat intestinal sucrose, maltose, and porcine pancreatic alpha-amylase [108]. The ethanolic extract of the leaves exhibited hepatoprotective effects against H_2_O_2_- and CCl_4_-induced liver damage [108].

### 3.17. Ocimum canum L.

#### 3.17.1. Traditional Uses

*O. canum* is native to tropical Africa [109].

*O. canum* is used for the treatment of various types of diseases, including lowering blood glucose [107]. The leaves of *O. canum* are used for the treatment of diabetes in Ghana [110,111].

#### 3.17.2. Phytochemical Constituents

The leaves of *O. canum* are rich in flavonoids and tannins [111]. Furthermore, the phytochemical screening of *O. canum* displayed anthocyanins, flavonoids, alkaloids, terpenes, and coumarins predominantly [110].

#### 3.17.3. Antidiabetic Activity

*O. canum* has been reported to inhibit the growth of cataracts in diabetic patients. Aqueous extracts of the leaves showed anti-hyperglycaemic activity [111].

The total extract demonstrated a significant (*p* 0.01) decrease in blood glucose levels and ameliorated other altered biochemical parameters which were related to diabetes. Moreover, histopathological modifications of the pancreas were also observed in streptozotocin-induced diabetic rats [96].

### 3.18. Rosmarinus officinalis L.

#### 3.18.1. Traditional Uses

*Rosmarinus officinalis* is native to the Mediterranean region, and it is one of the most popular evergreen culinary herbs cultivated worldwide, including in South America [112].

Rosemary has been traditionally used since ancient times to alleviate renal colic, analgesic, rheumatic, carminative, diuretic, expectorant, dysmenorrhea, asthma, colds, bronchitis, flu, digestive, palpitation, anaemia, dizziness, pain, anxiety soothing hearth, hypertension, insomnia, sluggishness memory, labyrinthitis, vitiligo, tachycardia, and diabetes [113,114,115].

#### 3.18.2. Phytochemical Constituents

Rosemary has been reported to contain various classes of polyphenols, such as flavonoids, phenolic acids, and phenolic terpenes [114]. Carnosol, carnosic, and rosmarinic acids have been reported to be the most abundant constituents [113,114,115,116].

Several classes of compounds have been isolated, including flavonoids (diosmetin, diosmin, genkwanin, hispidulin, luteolin, rutin, genkwanin, kaempferol-3-*O*-rutinoside, kaempferol, naringenin-*C*-hexoside, hesperetin, apigenin-7-*O*-glucoside, quercetin, and apigenin); terpenoids such as triterpenes (oleanolic, ursolic, and betulinic acids); diterpenes (carnosic acid, carnosol, methoxycarnosol, epirosmanol, rosmanol, isorosmanol, rosmadial, rosemaridiphenol, and methoxycarnosate); polyphenols (caffeic, chlorogenic, labiatic, neochlorogenic, and rosmarinic acids; coumaric acid; *m*-hydroxybenzoic acid; coumaroylquinic acid; vanillic acid; ferulic acid; syringic acid; protocatechuic acid, dicaffeoylquinic acid; and hydroxyphenylacetic acids: homovanillic acid and *p*-hydroxybenzoic acid) [10].

#### 3.18.3. Antidiabetic Activity

Rosemary extract and its polyphenols (carnosic and rosmarinic acids) have been reported to possess significant antidiabetic effects in different in vivo models of type 2 diabetes and insulin-like effects in insulin target cells in in vitro models [117].

The aqueous extract has been reported to potentially reduce the oxidative stress induced by streptozotocin and blood glucose levels [118]. Rosemary was found to demonstrate significant alpha-glucosidase inhibitory activity (60% decreases) [114].

### 3.19. Salvia lavandulifolia Valh

#### 3.19.1. Traditional Uses

*Salvia lavandulifolia* is native to the Iberian Peninsula. It is widely distributed in the Mediterranean area, mainly from the east of Spain to the Western Mediterranean, south east France, and north west Africa (Morocco and Algeria) [119].

*S. lavandulifolia* is traditionally used to treat diabetic hyperglycaemia [119].

#### 3.19.2. Phytochemical Constituents

The main phytochemical constituents of the aerial parts of *S. lavandulifolia* are flavonoids and terpenoids. Diterpenoids are the main compounds found in the roots. The herb presents phenolic monoterpenoids, flavones, and rosmarinic acid [119]. Numerous compounds, such as ursolic acid and galdosol, were reported from the same source [120].

#### 3.19.3. Antidiabetic Activity

The bio-evaluation of the hypoglycaemic activity of *S. lavandulifolia* demonstrated that this plant significantly decreases the blood glucose levels in alloxan-diabetic rabbits [121].

### 3.20. Salvia officinalis L.

#### 3.20.1. Traditional Uses

*Salvia officinalis* is native to the Southern Europe and Mediterranean areas; it is a perennial round shrub widely naturalized throughout the world [122].

*S. officinalis* is used against diabetes mellitus in many countries [123]. *S. officinalis* is used in Asia and Latin America’s folk medicine to treat different kinds of diseases, such as seizure, gout, ulcers, rheumatism, dizziness, inflammation, tremor, diarrhoea, paralysis, obesity, and diabetes [124].

#### 3.20.2. Phytochemical Constituents

The major phytochemicals found in the flowers, leaves, and stems of *S. officinalis* are fatty acids, carbohydrates, glycosidic derivatives (flavonoid glycosides, cardiac glycosides,), phenolic compounds (coumarins, tannins, flavonoids), polyacetylenes, steroids, terpenoids (monoterpenoids, diterpenoids, triterpenoids, sesquiterpenoids), and waxes [123]. Chlorogenic and ellagic acids, epigallocatechin gallate, epicatechin, rutin, luteolin-7-glucoside, quercetin, and rosmarinic acid are the most predominant compounds [125].

#### 3.20.3. Antidiabetic Activity

*S. officinalis* has been reported to have a wide range of pharmaceutical applications, including hypoglycaemic and hypolipidemic effects. Additionally, *S. officinalis* has been reported to have a hypoglycaemic effect on diabetic animals and be beneficial for type 2 diabetic patients due to its ability to reduce liver glucose production [123,126]. The methanolic extracts of *S. officinalis* have considerably decreased serum glucose levels in type 1 diabetic rats. The aqueous extract of *S. officinalis* has been found to possess insulin-like effects [123].

Infusions (tea) of *S. officinalis* have been reported to reduce liver glucose production and increase insulin action. *S. officinalis* has been demonstrated to be as powerful as metformin, a well-known oral antidiabetic drug utilized for the treatment of type 2 diabetes [123].

### 3.21. Salvia fruticosa Mill

#### 3.21.1. Traditional Uses

*Salvia fruticosa* is native to the Eastern Mediterranean area and Jordan [15].

*S. fruticosa* has also been utilized for improving memory and as a hypoglycaemic agent [127].

#### 3.21.2. Phytochemical Constituents

Three flavonoids named luteolin, apigenin, and rutin, in addition to three phenolic acids named ferulic, gallic, and rosmarinic acids, were identified from the aerial parts. Moreover, dehydro-abietic acid and carnosol were isolated from the root [128].

#### 3.21.3. Antidiabetic Activity

*S. fruticosa* has been reported to possess hypoglycaemic activity by reducing the intestinal absorption of glucose [129]. This plant is well known for its antidiabetic activities in Jordan. The oral administration of a 10% leaf infusion of 0.25 g/kg BW caused a significant reduction in blood glucose levels in alloxanized rabbits without exerting any effect on normal ones [15].

### 3.22. Teucrium polium L.

#### 3.22.1. Traditional Uses

*Teucrium polium* is native to Southwest Asia and the Mediterranean region. It widely distributed in nearly all the Mediterranean countries, Europe, south-western Asia, and North Africa [130].

*T. polium* is traditionally used for the treatment of different kinds of pathological conditions, such as diabetes, inflammations, gastrointestinal disorders, and rheumatism [131]. It is also used by Iranians for its anti-inflammatory, antipyretic, diuretic, diaphoretic, tonic, antispasmodic, antihypertensive, analgesic, antibacterial, and antidiabetic effects [132]. In southern Iran, many type 2 diabetic patients use the aqueous extract made from the dried aerial parts of *T. polium* as an antidiabetic drug [130].

#### 3.22.2. Phytochemical Constituents

Phytochemical investigations of *T. polium* have revealed that various classes of compounds such as terpenoids, flavonoids, iridoids, and sterols are present in its aerial part and root. Numerous flavonoids such as salvigenin, luteolin, apigenin, cirsiliol, rutin, cirsimaritin, and eupatorin have been reported from the roots and aerial parts. Additionally, two iridoid glycosides, teuhircoside and teucardoside, have been isolated from a hydrophilic fraction [130]. Several steroidal compounds, such as stigmasterol, β-sitosterol, campesterol, clerosterol, and brassicasterol, have also been isolated from the same source [133].

#### 3.22.3. Antidiabetic Activity

*T. polium* and its isolates have been reported to have a broad spectrum of pharmacological applications, including hypoglycaemic and hypolipidemic effects. *T. polium* enhanced insulin secretion by nearly 135% after a single dose of the plant extract (equivalent to 0.1 mg plant leaf powder per mL of the culture medium) at a high glucose concentration (16 mmol/L). Its aqueous extract (50 mg/kg) significantly (*p* 0.05) decreased the serum glucose levels of diabetic Sprague–Dawley male rats from 283.622.1 to 96.211.9 mg/dL [130].

*T. polium* extract has been reported to reverse the symptoms of streptozotocin-induced diabetes in rats by adjusting the pancreatic transcription factor pancreas/duodenum homeobox gene-1 (Pdx1) and forkhead transcription factor (FoxO1) expressions [134].

*T. polium* showed a considerable decrease in the blood glucose level of STZ-diabetic rats and demonstrated protective effects on pancreatic tissue in STZ-induced oxidative stress based on its strong oxidative capacity. Furthermore, *T. polium* showed weak alpha-amylase inhibitory activity (5%) [15].

### 3.23. Teucrium cubense Jacq

#### 3.23.1. Traditional Uses

*Teucrium cubense* is native to northern and tropical Africa, and it is widely distributed in Coastal Germander, sandy clay, Padre, and the Matagorda Islands [135].

*T. cubense* is considerably employed in the Mexican folklore to treat type 2 diabetes. It is used as an oral hypoglycaemic agent in Saudi Arabia and North Africa [15].

#### 3.23.2. Phytochemical Constituents

Numerous neo-clerodane diterpenes, abietane diterpenes, sesquiterpenes, triterpenes, and flavonoids have been isolated from *T. cubense* [135].

#### 3.23.3. Antidiabetic Activity

The aqueous extract of *T. cubense* has been reported to decrease plasma glucose levels in healthy rabbits. Additionally, 70 µg/mL of *T. cubense* extract activated glucose uptake by 112% (murine) and 54% (human) in insulin-sensitive cells. At the same time, it induced the incorporation of glucose by 69% (murine) and 31% (human) in insulin-resistant adipocytes [135].

According to the scientific databases consulted for this review, twenty-three plant species of the Lamiaceae family, belonging to twelve (12) genera, are used for managing and treating diabetes mellitus worldwide (Table 1). Table 1 also provides relevant information regarding the plant species, part of the plant used, mode of preparation, and geographic locations of their traditional uses for diabetes.

## 4. Antidiabetic Activity of Some Notable Chemical Constituents Isolated from Lamiaceae Species

Numerous classes of compounds have been reported to be the bioactive constituents of numerous plant species of the Lamiaceae family used in folk medicine for their potential applications in the management of diabetes and related complications. Among these bioactive compounds, flavonoids such as rutin, luteolin, apigenin, and salvigenin have demonstrated significant antidiabetic activity in different models (in vitro and in vivo), due to the existence of the hydroxyl group allocated at C-3 on the A-ring and other hydroxyl groups attached to the C-ring, which play an essential role in the inhibition process of alpha-glucosidase and-amylase enzymes as well as hypoglycaemia, insulin activation, and glucose uptake activation [136]. On the other hand, various classes of compounds such as hydroxycinnamic acids (caffeic and rosmarinic acids), diterpenes (forskolin and marrubiin), and triterpenes (ursolic and oleanolic acids, 16-hydroxy-4,4,10,13-tetramethyl-17-(4-methyl-pentyl)-hexadecahydro-cyclopenta[a]phenanthren-3-one have also exhibited prominent antidiabetic properties with a different mode of action, as illustrated in Table 2. The hypoglycaemic effects of numerous compounds such as luteolin, apigenin, rosmarinic, caffeic, ursolic, oleanolic, and chlorogenic acids have been described in Table 2, in addition to compounds such as rutin, quercetin, and forskolin that have been reported to stimulate β-cells to release more insulin. Furthermore, marrubiin has been reported to increase the level of insulin and glucose transporter-2 gene expressions in INS-1 cells [30,33,63,93,94,96,137,138,139,140,141,142,143,144,145].

## 5. Conclusions

**Figure 1:**
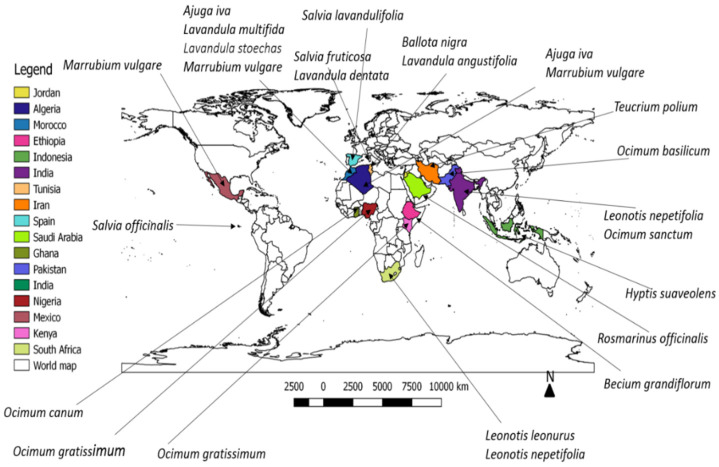
Plants species in the Lamiaceae family that are traditionally used to treat diabetes in different countries.

This comprehensive review shows that the Lamiaceae species is used traditionally to treat diabetes by peoples of different regions and continents, including Africa, Asia, South and Central America, the Middle East, and Europe (Figure 1). Remarkably, four of the twenty-three species presented in this paper are used to manage diabetes in Morocco; meanwhile, most of the species—nine of twenty-three species—used for the management of diabetes belong to only two genera, *Lanvandula* and *Ocimum*. Additionally, this review highlights the antidiabetic capabilities and pharmacological mechanisms of action of each plant extract and some notable chemical constituents isolated from some of these Lamiaceae species. The scientific validation of the traditional use of Lamiaceae species in managing and preventing diabetes and related complications is presented.

Based on the literature reviewed, the Lamiaceae species is a potential source of antidiabetic agents. However, further research studies based on preclinical and clinical studies are required to clarify the use of the Lamiaceae species in the management of diabetes, emphasizing its potential therapeutic application in the prevention of diabetes and related complications. Moreover, ethnobotanical, preclinical, and clinical investigations will contribute towards developing, promoting, and managing indigenous knowledge systems.

## Figures and Tables

**Table 1:** List of plants used traditionally for diabetes.

Plant Species	Part of the Plant	Mode of Preparation	Geographic Location	Antidiabetic Activity	References
*Ajuga iva* (L.) Schreb	Aerial parts (Leaf and stem)	Decoction, infusion, and raw Powder	Morocco and Algeria	Hypoglycaemic, hypolipidemic, alpha-glucosidase and alpha-amylase activities	[13,14,17,19,20]
*Ballota nigra* L.	*Aerial part*			Hypoglycemic activity	[21]
*Becium grandiflorum* (Lam.) Pic.Serm	Leaves	Powder	Ethiopia	Antihyperglycemic activity	[24,25]
*Calamintha officinalis* Moench	Stem, leaves, and seeds	Decoction (aqueous)	Morocco	Hypoglycaemic activity	[28]
*Coleus forskohlii* (Willd.) Briq.	All plant		West Africa and India	Hypoglycaemic activityWeight-loss	[33,35]
*Hyptis suaveolens* (L.) Poit.	Aerial part		Indonesia	Antihyperglycemic activity	[41,43]
Lavandula angustifolia Mill.	Aerial part		Jordan	Inhibition of hormone sensitive lipase and pancreatic lipase activities	[45]
*Lavandula dentata* L.	Leaves and stem	Decoction, infusion, and raw	Mediterranean or Saharan regions	Hypolipidemic and hypoglycaemic activities	[51,54]
*Lavandula multifida* L.	leaves and stems	decoction	Morocco	Antihypolipidemic and hypoglycaemic activities	[55,56,58,60]
*Lavandula stoechas* L.	Leaves	Decoction	Morocco, Tunisia	Hypoglycaemic activity	
*Leonotis leonurus* (L.) R. Br.	Leaves and stems		South Africa	Antihyperglycemic, hypoglycaemic and antilipidemic activities	[63,64,66]
*Leonotis nepetifolia* (L.) R.Br.	Leaves		South Africa, India	Antidiabetic activity	[68,69,72]
*Marrubium vulgare* L.	Leaves	Decoction	Morocco, Mexico, and Algeria	Hypoglycaemic, antihyperlipidemic, alpha-glucosidase and alpha-amylase activities	[74,75,78,80]
*Ocimum gratissimum* L.	Leaves	Infusion, food vegetable	Western Africa, Nigeria	Antihyperglycemic and hypoglycaemic activities	[86,96]
*Ocimum sanctum* L.			India	Hypoglycaemic activity	[98,100]
Ocimum basilicum L.			Pakistan, Asia	Hyperlipidemia and hypoglycaemic activities	[103,104,106]
*Ocimum canum* L.	Leaves		Ghana	Antihyperglycemic activity	[109,111]
*Rosmarinus officinalis* L.			Kingdom of Saudi Arabia	Alpha-glucosidase and hypoglycaemic activities	[114,115]
*Salvia lavandulifolia* Valh	Leaves		Spain	Hypoglycaemic activity	[119,121]
*Salvia officinalis* L.	Leaves	Decoction and infusion	Asia, Latin America	Hypoglycaemic and hypolipidemic effects	[123,126]
*Salvia fruticosa* Mill.	Aerial part	Tea	Jordan	Hypoglycaemic activity	[15,127]
*Teucrium polium* L.	Dried aerial parts	Decoction and powder	South Iran	Hypoglycaemic activity	[130,131,132,134]
*Teucrium cubense* Jacq.			Mexican, Saudi Arabia, and North Africa	Hypoglycaemic activity	[15,135]

**Table 2:** Antidiabetic activity of different constituents of the Lamiaceae species.

Compounds	Plant Source	Biological Activity/Mode of Action	References
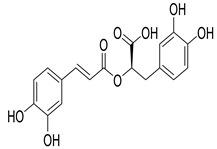 Rosmarinic acid	*C. officinalis* *Rosemary* *S. lavandulifolia* *O. canum*	Hypoglycaemic effect Significant antidiabetic effects in different in in vivo models and insulin-like effects in insulin target cells in in vitro models of type 2 diabetes	[30,63]
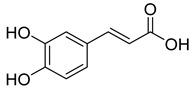 Caffeic acid	*C. officinalis* *S. officinalis*	Hypoglycaemic effect	[30]
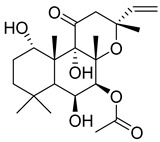 Forskolin	*C. forskohlii*	Glucose-induced insulin secretion Decreases fasting blood glucose levels Enhances the glucose-mediated stimulus Induces cells to release insulin Decreases basal glucose in healthy rats Attenuates the severity of hyperglycaemia in diabetic Rats	[33]
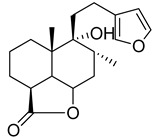 Marrubiin	*L. leonurus*	Increases the level of insulin and glucose transporter-2 gene expressions in INS-1 cells	[137]
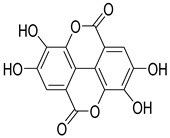 Ellagic acid	*S. officinalis*	Stimulates insulin secretion and decreases glucose intolerance	[138]
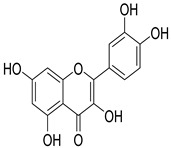 Quercetin	*S. officinalis*	Stimulate β-cells to release more insulin	[139]
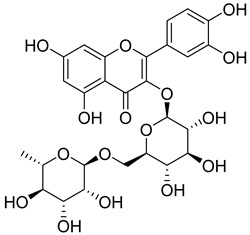 Rutin	*S. officinalis*	Stimulates β-cells to produce more insulin	[139]
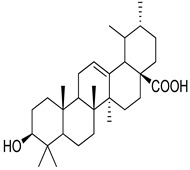 Ursolic acid	*S. officinalis*	Hypoglycaemic effect Stimulate glucose uptake	[140,141]
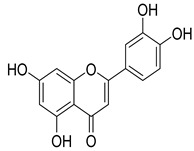 Luteolin	*T. polium*	Antidiabetic effects and hypoglycaemic effect	[142]
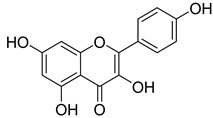 Apigenin	*T. polium*	Reduces blood glucose The hypoglycaemic effect in diabetic rats Stimulate the synthesis of glycogen in muscles Antihyperglycemic and Insulinmimetic activities	[143]
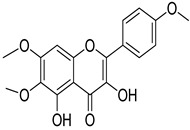 Salvigenin	*T. polium*	Improves diabetes through decreasing blood glucose, lipid profile, HbA1c. Increased insulin secretion	[144]
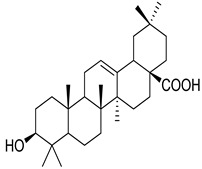 Oleanolic acid	*H. suaveolens*	Improves insulin response Significant blood glucose-lowering and weight loss effect	[145]
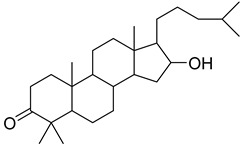 16-hydroxy-4,4,10,13-tetramethyl-17-(4-methyl-pentyl)-hexadecahydro-cyclopenta[a]phenanthren-3-one	*O. sanctum*	Antihyperglycemic activity	[96]
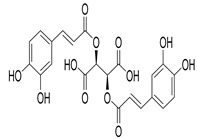 Chicoric acid	*O. gratissimum*	Significant decrease in the glycemic levels in diabetic mice	[93]
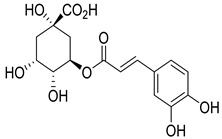 Chlorogenic acid	*O. gratissimum*	Significant hypoglycaemic activity in streptozotocin-induced diabetic rats	[94]

The stereochemistry of 16-hydroxy-4,4,10,13-tetramethyl-17-(4-methyl-pentyl)-hexadecahydro-cyclopenta[a]phenanthren-3-one was not reported on the original article.
